# The Role of CDK5 in Tumours and Tumour Microenvironments

**DOI:** 10.3390/cancers13010101

**Published:** 2020-12-31

**Authors:** Phuong Anh Do, Chang Hoon Lee

**Affiliations:** Phamaceutical Biochemistry, College of Pharmacy, BK21 FOUR Team, and Integrated Research Institute for Drug Development, Dongguk University, Goyang 100-715, Korea; dophuonganh@dgu.ac.kr

**Keywords:** CDK5, cancer, neuron, microtubule, tumour microenvironments

## Abstract

**Simple Summary:**

CDK5 is a serine/threonine type kinase that is mainly found in nerves. It is a target that has been studied primarily in neurological disorders, but recently it has been newly recognised for its importance in cancer. In this review, we mentioned the role of CDK5 in normal cells and the latest findings that CDK5 contributes to ten hallmarks of cancer and cancer-nerve connections. Also, we introduced representative CDK5 inhibitors and suggested the possibility of CDK5 inhibitors as treatments for refractory cancer.

**Abstract:**

Cyclin-dependent kinase 5 (CDK5), which belongs to the protein kinase family, regulates neuronal function but is also associated with cancer development and has been proposed as a target for cancer treatment. Indeed, CDK5 has roles in cell proliferation, apoptosis, angiogenesis, inflammation, and immune response. Aberrant CDK5 activation triggers tumour progression in numerous types of cancer. In this review, we summarise the role of CDK5 in cancer and neurons and CDK5 inhibitors. We expect that our review helps researchers to develop CDK5 inhibitors as treatments for refractory cancer.

## 1. Introduction

Cyclin-dependent kinase 5 (CDK5), a proline-directed serine/threonine kinase, is known as a modulator of neuron function, including neurite outgrowth, neuron migration, and neuron degeneration [1,2,3,4,5,6]. Recently, CDK5 has been proposed to play a vital role in cancer development, and the overexpression of CDK5 correlates with poor prognosis, tumour proliferation, migration, and invasion in a variety of cancers [7,8,9,10,11]. Hence, CDK5 regulation is a potential cancer therapeutic target. In this review, we summarise the normal function of CDK5, its role in cancer development, a potential CDK5-mediated tumorigenesis pathway, and potential therapeutic options. We hope that this review can provide a reliable platform for future research about CDK5 as a target for cancer treatment.

## 2. Biology of CDK5

CDK5 was first identified by Hellmich in 1992 as neuronal cell division control 2-like kinase due to its high sequence homology of the cell division cycle protein 2 (cdc2) [12]. It has 292 amino acids and around 5000 nucleotides. CDK5 is expressed in mammalian tissue and culture cells, and it is co-localised with its substrates and activators [13,14]. Other cyclin-dependent kinases require the phosphorylation on the T loop, but the binding of subunits is sufficient for the activation of CDK5 [15]. However, it seems that the phosphorylation of Ser159 on the T loop of CDK5 and the binding of p35 are necessary to exhibit maximum activity.

### 2.1. Basics of CDK5, Its Activators, and Inhibitors

Unlike other CDK family members that require cyclin for activation [16], CDK5 mainly binds to p35 or p39 or their truncated products to convert to the active form (Table 1) [17,18,19]. CDK5 can also be activated through binding to cyclin I in both neurons and podocytes [20]. In contrast, some cyclin proteins, such as cyclin D1, cyclin E, and glutathione S-transferase P, can inhibit the activity of CDK5 (Table 1) [21,22].

#### 2.1.1. p35

p35, a membrane–docked protein, consists of two parts: an N-terminal region containing a p10 component and a C-terminal region containing p25 [33]. The p10 component encompasses a myristoylation sequence to localise p35 to the phospholipid membrane [24]. Moreover, p10 is the signal area for p35 degeneration. Hence, p35 is an unstable protein with a short half-life [3,34,35]. Although p35 binds to the membrane through myristoylation, p35 is also found in the nuclei of neuronal and non-neuronal cells [36]. The transport of p35 into nuclei is mediated through its interaction with importins [37]. This importation leads to the disassociation of CDK5 from p35 [37].

The activity of p35 can be modulated by nerve growth factor (NGF) and brain-derived neurotrophic factor (BDNF). NGF treatment in PC12 cells facilitates strong induction of p35 expression via the activation of the extracellular-signal-regulated kinase (ERK) pathway [38]. In a neuron, phosphatidylinositide 3-kinase (PI3K) is the target of BDNF. The BDNF-mediated activation of PI3K leads to an increase in the level of p35 [39]. Interestingly, CDK5 can phosphorylate p35 at Ser^8^ and Thr^138^ [40,41]. The phosphorylation of Ser^8^ diffuses cytoplasmic localisation [41]. This may be due to reduced interaction between p35 and membrane phospholipid, which increases the mobility of p35 on the membrane [41]. The phosphorylation of p35 at Thr^138^ interferes with calpain to convert p35 into p25 [40].

#### 2.1.2. p25

**Figure 1:**
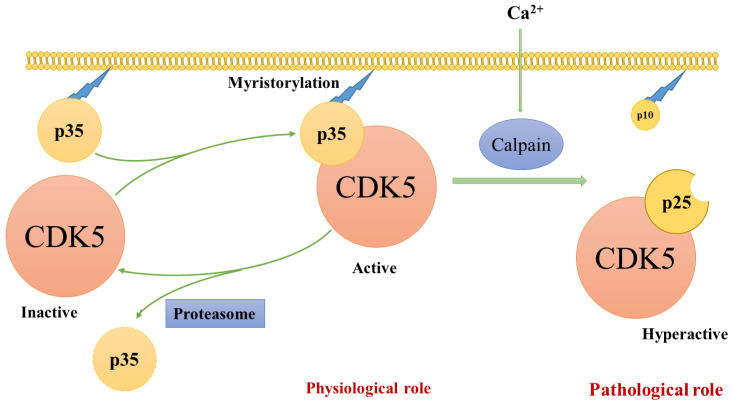
The mechanism of Cdk5 activation. The mechanism of Cdk5 activation. CDK5 alone is an inert catalyst subunit. CDK5 is activated by the p35 CDK5 activator and moves to the membrane as p35 binds to the membrane through myristoylation of the N-terminal region. p35 is a short-lived protein that is broken down by proteasomes. When cells are stressed or met with death signal, calpain is activated and cuts p35 into p25 C-terminal fragments. The deletion of p10 prolongs the half-life of p25. CDK5/p25 can be separate from the membrane and phosphorylate additional proteins. (modified from Kimura et al. [46]).

CDK5 can be regulated by p25, the truncated product of p35 (Table 1) [19,24]. Under oxidative stress conditions, intracellular neuronal Ca^2+^ homeostasis is disrupted, resulting in the activation of calpain [24,42]. Calpain cleaves p35 into p10 and p25, containing the binding domain to CDK5 (Figure 1) [43,44,45]. Compared with p35, p25 is resistant to ubiquitin-mediated degradation, so it is more stable and has a longer half-life [24,43,44]. This results in an extended CDK5 activation period, inducing the hyperphosphorylation of CDK5 target molecules and neuronal toxicity [24]. For example, abnormal phosphorylation of protein tau caused by CDK5/p25 complexes leads to microtubule instability and the formation of the neurofibrillary tangles that characterise Alzheimer’s disease [24,46,47].

The presence of p10 helps to localise CDK5/p35 mainly in the perinuclear and plasma membrane regions and less prominently in the nucleus-the cleavage of p10 from p35 by calpain yields p25. Thus, CDK5/p25 complexes are enriched in the cytosolic region and nucleus [23,24]. However, the p10 region of p35 can move into the nuclei when myristoylation does not occur [23]. The mechanism underlying the translocation of CDK5 and its activators into the nuclei remains unclear.

#### 2.1.3. p39 and p29

p39 is an isoform of p35 and an activator of CDK5; it also has the same position as p35 (Table 1) [41,48]. Similar to p35, p39 includes p10 with a myristoylation site and p29 [23,48]. Thus, the calpain-mediated conversion of p39 into p29 ensues in the same manner as the cleavage of p35 into p25 [23]. However, unlike p35, p39 contains a small insertion (amino acids 329–366) that allows it to bind to muskelin to promote cell adhesion [49].

Like p35, p39 is also phosphorylated by CDK5 at Ser^8^, leading to localisation in the cytoplasm [41]. However, because of the Lys cluster in the p10 region of p39, p39 presents stronger nuclear localisation rather than p35 [41]. CDK5-mediated Thr84 phosphorylation of the C–terminal region of the Lys cluster in p39 attenuates stronger nuclear localisation ability of p39 [41].

#### 2.1.4. Other Activators and Inhibitors

Cyclin I is found at differentiated podocytes and neurons along with CDK5 (Table 1) [26]. Moreover, cyclin I is capable of binding and activating CDK5 to form cyclin I-CDK5 complexes, which in turn regulate the levels of pro-survival proteins B-cell lymphoma 2 (Bcl-2) and B-cell lymphoma-extra large (Bcl-XL) through facilitating the mitogen-activated kinase (MEK)-ERK pathway [26,50]. This prevents the apoptosis of post-mitotic cells [26].

Evidence indicates that cyclin D1 fails to stimulate CDK5 activity [28]. Abnormal activation of the MEK-ERK pathway by neurotoxicity enhances the expression of cyclin D1, subsequently causing neuronal cell cycle re-entry and neuronal apoptosis [28]. Moreover, cyclin D1 prevents the interaction between CDK5 and p35, leading to the downregulation of CDK5/p35 complex (Table 1) [28]. This, in turn, causes hyperactivation of the MEK-ERK pathway and programmed cell death of neurons [28].

Cyclin E is expressed in neuron and forms complex with CDK5 (Table 1) [30]. Like cyclin D, cyclin E also inactivates CDK5 via the dissociation of CDK5 from its activators, p35 and p39 to modulate the formation of synapses [30]. Also, the deletion of cyclin E causes defective synaptic plasticity and memory deficits [30].

Glutathione-S-transferase P (GSTP1) inhibits the activity of CDK5 by dislodging p25 and p35 from CDK5/p25 and CDK5/p35 complexes, respectively (Table 1) [22]. Even in the context of high p25 and p35 levels, GSTP1 upregulation is associated with decreased CDK5 activity [22]. GSTP1 also indirectly inhibits the activation of CDK5 by eliminating oxidative stress [22].

Munc 18 (p67) is part of a multimeric (supramolecular) complex containing Cdk5/p35 and regulates CDK5 activity (Table 1) [51,52]. Evidence showed that Munc18 could protect CDK5/p35 complex from TFP5, which can exert an inhibitory effect on CDK5 activity [31,53]. However, the effect of TFP5 on CDK5/p25 complex is not affected by Munc18 [31]. This could be explained by Munc18’s ability to bind to p10 component of p35, which is lacking in p25 [31].

### 2.2. CDK5 Regulation by Posttranslational Modification

CDK5 can be controlled by posttranslational modifications, including phosphorylation, S-nitrosylation, and acetylation (Table 2). There is evidence that proto-oncogene tyrosine-protein kinase Fyn, c-Abl, Eph receptor A4, and tropomyosin receptor kinase B can phosphorylate CDK5 at Tyr^15^ to enhance the activity of CDK5 [54,55,56,57].

This ensues in neurite and spine retraction, dendrite outgrowth, and neuron death, suggesting the importance of the phosphorylation of CDK5 at Tyr^15^ [54,55,56,57,66]. However, the phosphorylation Tyr^15^ of CDK5 occurs with monomeric CDK5 but not with CDK5/p35 complexes [67]. Interestingly, tyrosine kinase can upregulate p35 to activate CDK5 [67].

To achieve the maximal activation, CDK5 requires binding to p35 and phosphorylation at Ser^159^ [58]. Similar to this, an enzyme-containing CDK7 can facilitate the activity of CDK5/p25 complexes through the phosphorylation Ser^159^ of CDK5, leading to pathological events [59].

The phosphorylation Thr^14^ of CDK5 inhibits CDK5 kinase activity [68]. This phosphorylation can cause ATP phosphate moiety misalignment and changes in the hexacoordinated sphere of the Mg^2+^ [68]. The ablation of cub-domain containing protein-1 facilitates the proto-oncogene tyrosine-protein kinase Src (c-Src)–mediated phosphorylation of p35, which in turns binds to protein kinase C-delta (PKCδ) [62]. Subsequently, PKCδ phosphorylates CDK5 at Thr^77^ to sequester CDK5 away from p35 and restrain CDK5 kinase activity [62]. Moreover, CDK5 is regulated by S-nitrosylation at Cys^83^ and Cys^157^, leading to a reduction in CDK5 activity [63,64]. The ablation of S-nitrosylation of CDK5 promotes dendritic growth [64]. This indicates the role of CDK5 S-nitrosylation in neuronal development. Moreover, the acetylation-mediated modulation of CDK5 activity influences neurite outgrowth in hippocampal neurons [65]. The acetylation of nuclear CDK5 at Lys^33^ restrains its kinase activity by losing ATP binding [65].

### 2.3. Regulation of Transcription and Translation by CDK5

CDK5 is located in both the nucleus and cytoplasm. It can phosphorylate several substrates resulting in the regulation of transcription and translation. Forty-nine per cent of CDK5 substrates contain a consensus sequence (Ser/Thr)-Pro-X-(Arg/Lys/His) [69].

#### 2.3.1. Transcription Regulation

CDK5 has been demonstrated as a critical transcriptional modulator of gene expression through the direct and indirect phosphorylation of transcription factors (Table 3). Myocyte enhancer factor 2 (MEF2) is a major transcription factor associated with muscle development [70]. CDK5/p25 complexes can phosphorylate MEF2, resulting in inhibition of the pro-survival transcription function of MEF2 [71]. Consistent with this, nuclear CDK5 phosphorylates MEF2 at Ser^444^ to block MEF2 activity and facilitate the caspase-mediated degradation of MEF2 [72,73].

Signal transducer and activator of transcription 3 (STAT3) is phosphorylated by CDK5 at Ser^727^ [74]. This phosphorylation controls cell proliferation in medullary thyroid and prostate cancer [75,76]. Although the phosphorylation Tyr^705^ is sufficient for the activation of STAT3, Ser^727^ phosphorylation is required for the maximal transcription activity of STAT3 [77]. Notably, the expression of c-fos and jun B and the transcription activity of STAT3-targeted genes, are attenuated by the blockade of CDK5 activity [77].

Evidence shows that CDK5/p35 or CDK5/p25 complex can bind to phosphorylate mineralocorticoid and glucocorticoid receptors, subsequently modulating their transcriptional activity and neuronal functions [78,79]. CDK5/p25 complexes phosphorylate p53, contributing to the enhanced expression of p53, the transcription activity of TP53, and the p53 target gene, p21 [80]. Similarly, Lee et al. have demonstrated that CDK5 can phosphorylate p53 at Ser^15^, Ser^33^, and Ser^46^ to stabilise and accumulate nuclear p53 [81]. This facilitates the transcriptional activity of p53, leading to neuronal cell death [81]. Under hyperosmotic conditions, CDK5 phosphorylates tonicity-responsive enhancer-binding protein/osmotic response element-binding protein (TonERP/OREBP) at Thr^135^ to accumulate TonEBP/OREBP in the nucleus and to promote its transcription of osmoprotective target genes [82]. CDK5/p35 phosphorylates mitogen-activated protein kinase kinase-1 (MEK1) at Thr^286^, leading to the inhibition of cAMP response element-binding protein (CREB) transcriptional activity, which in turn causes apoptosis [83]. Mouse Sds3 (mSds3) can be negatively regulated via CDK5-mediated phosphorylation [84]. The interaction between mSds3 and p35 allows CDK5 to phosphorylate mSds3 [84]. This regulates mSds3 homodimerisation and suppresses m-Sin3-dependent transcription, subsequently affecting neuron and muscle development [84].

#### 2.3.2. Translation Regulation

**Figure 2:**
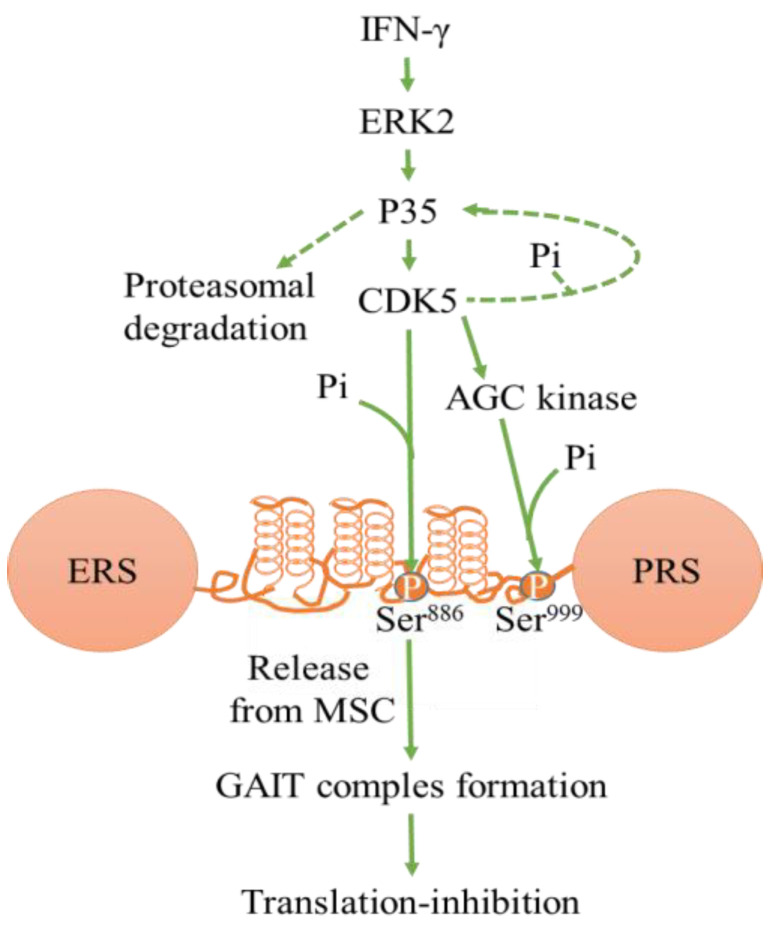
Schematic of kinase pathway phosphorylating EPRS Ser886 and Ser999. IFN-γ–activated CDK5 phosphorylate EPRS, leading to the formation of the GAIT complex. This inhibits inflammatory mRNA translation. Abbreviations- interferon-gamma (IFNγ), glutamyl-prolyl tRNA synthetase (EPRS), IFN-γ–activated inhibitor of translation (GAIT), tRNA multisynthetase complex (MSC). (modified from Arif et al. [85]).

CDK5 can regulate translation (Figure 2). Through p35 and ERK2, interferon-gamma (IFNγ) activates CDK5 to phosphorylate glutamyl-prolyl tRNA synthetase (EPRS) at Ser^886^, subsequently phosphorylating Ser^999^ of EPRS in myeloid cells [85]. The process contributes to the translocation of EPRS from tRNA multisynthetase complexes (MSC) into NS1-associated protein (NSAP1) to form pre- IFN-γ–activated inhibitor of translation (GAIT) complexes [85]. This, in turn, binds to phospho-ribosomal protein L13a and glyceraldehyde-3-phosphate dehydrogenase (GAPDH) and inhibits inflammatory mRNA translation [85].

## 3. Role of CDK5 in Normal Cell Physiology

CDK5 is associated with cell physiology through its effects on cell adhesion, the cytoskeleton, the cell cycle and DNA damage [86,87,88].

### 3.1. CDK5 and Cell Adhesion

**Figure 3:**
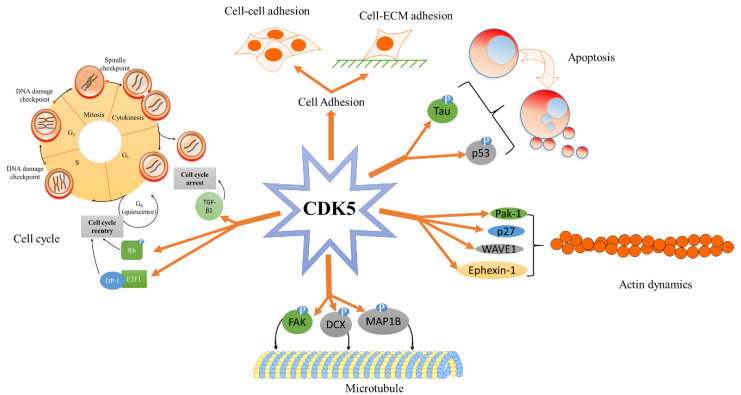
A summary of the various cyclin-dependent kinase 5 (CDK5)-mediated biological processes. CDK5 plays important roles not only in the central nervous system but also in different biological processes. Functions in the central nervous system include synaptic function, axon guidance, cell adhesion, and neurodegenerative diseases. Functions outside of the central nervous system include androgen production, cell cycle, cancer cell proliferation/apoptosis, and tumour metastasis. Abbreviations- microtubule-associated proteins 1B (MAP1B), focal adhesion kinase (FAK), doublecortin (DCX), p21 activated kinase 1 (Pak-1), Wiskott-Aldrich syndrome protein-family verprolin homologous protein 1 (WAVE-1), Eph receptor A4 (EphA4), transforming growth factor-β1′ (TGF-β1), retinoblastoma (Rb), E2F transcription factor 1 (E2F1). (modified from Shupp et al. [19]).

Cell adhesion is classified into two broad categories: cell-extracellular matrix (ECM), and cell-cell adhesion, with the regulated adhesion molecules being integrin and cadherin, respectively [89,90]. Evidence indicates that CDK5 plays a role in regulating cell adhesion through cell adhesion receptors (Figure 3) [87]. While CDK5 silence or p35 inhibition contributes to increasing N-cadherin-mediated adhesion, CDK5 overexpression causes the loss of adhesion [87].

The adhesion inhibition may be a prerequisite for the migration of neuronal cells [87]. This suggests that through N-cadherin-dependent adhesion, CDK5 controls neuron migration. Interestingly, p35 could bind to E-cadherin and play a role in CDK5-independent regulation of precursor form of E-cadherin but not mature form [91].

When discussing the role of CDK5 in nonneuronal cells, one should not simply pay attention to the expression of CDK5, but the presence or absence of activators such as p35 and p39; the enzymatic activity of CDK5 should also be checked. It was reported that the expression of p35, an activator of CDK5, was observed in cell-cell adhesion in epithelial lens cells and survival studies of pancreatic β-cells [92,93,94]. Moreover, CDK5 has been reported to enhance cell-substrate attachment, whereas CDK5 upregulation reduces cell-cell adhesion in rabbit lens [95]. Besides, treatment with roscovitine, a CDK5 inhibitor, promotes cadherin-mediated adhesion but prevents adhesion of the cell to fibronectin through integrin in human keratinocytes [96]. Increases of CDK5 kinase activity is concomitant with an increase in cells adhering to fibronectin [97].

Moreover, CDK5 can phosphorylate the talin head domain at Ser^425^ to inhibit talin head’s interaction with E3 ubiquitin-protein ligase SMURF1, inhibiting the degradation of the talin head [98]. Through this pathway, CDK5 modulates cell adhesion and cell migration [98].

### 3.2. CDK5 and Cytoskeleton

The cytoskeleton consists of three elements: microtubule, intermediate filaments, and actin [99]. These polymers modulate morphological changes and movement of eukaryotic cells [99]. Evidence shows that CDK5 plays a vital role in cytoskeletal regulation, especially microtubule regulation, in controlling neuronal migration, neuritic growth, and synaptogenesis [100,101]. Recently, the effect of CDK5 on cytoskeletal elements has been expanded to non-neuronal cells such as cancer cells [102,103,104].

#### 3.2.1. CDK5—Microtubule

Via its existing microtubule-polymerizing function, p35 acts as a modulator of microtubule architecture [105]. The p10 component of p35 contains microtubule- and tubulin-binding domain [105]. That is, the microtubule polymer inhibits CDK5-p35 activity by blocking the interaction between p35 and CDK5. Interestingly, the CDK5-mediated phosphorylation of p35 promotes the microtubule-binding and polymerising activity of p35 [106]. Moreover, CDK5/p25 complex upregulates the phosphorylation of microtubule-associated proteins 1B (MAP1B), which is among the microtubule dynamics regulators [107]. However, this cannot occur when CDK5 binds to p35 [107].

Furthermore, Ser^732^ phosphorylation of focal adhesion kinase (FAK) by CDK5 plays an essential role in microtubule organisation and microtubule fork formation to facilitate neuronal migration [108]. In contrast, the overexpression of non-phosphorylatable mutant of FAK or a CDK5 insufficiency results in disorganised microtubule fork formation [108]. CDK5 phosphorylates doublecortin (DCX) at Ser^297^, leading to the decreased binding between DCX and microtubules and the polymerising effect of DCX [109]. This makes microtubule more dynamic, which promotes cell migration [109]. According to these findings, CDK5 may regulate microtubule dynamics to affect cell migration.

#### 3.2.2. CDK5—Intermediate Filaments

Neurofilaments (NFs) are a specific type of intermediate filament. NFs accumulation leads to neurodegeneration and related diseases [110]. CDK5 can phosphorylate NFs and control axonal movement and are involved in developing neuronal diseases [6].

Nestin, one of the intermediate filaments, is found in specific organs and cells, such as neuroepithelial progenitor cells, myoepithelial breast cells, and renal podocytes [111]. Moreover, the expression of nestin increases in neoangiogenic blood vessels, injury and cancer [111,112,113]. Additionally, the nestin insufficiency poses effects on focal adhesion and cell migration, especially in the context of cancer [111]. Nestin is phosphorylated by CDK5 at Thr^316^ residue to monitor the reorganisation and dynamics of nestin [114]. Interestingly, p35 can bind to nestin after treatment with roscovitine, suggesting that the interaction between p35 and nestin serves to regulate CDK5 activity [114]. Moreover, nestin modulates the conversation of p35 into p25, regulating the activity of CDK5 in differentiating myoblasts [115].

#### 3.2.3. CDK5—Actin Cytoskeleton

CDK5 and p35 co-localize with actin filaments in neurite terminals [116]. Moreover, another activator of CDK5, p39, has been found to co-localize with actin at growth cones of hippocampal neurons [25]. In this environment, p39 can bind to actin, and the disruption of actin cytoskeleton leads to changes in the localisation of p39 and the activity of CDK5/p39 complex [25]. CDK5 regulates actin dynamics through interactions with actin regulatory proteins (Figure 3). For example, in a neuron, p35 can directly bind to Rac Family Small GTPase 1 (Rac-1) in a GTP-dependent manner, which forms a complex with CDK5 [117]. This causes the downregulation of p21 activated kinase 1 (Pak-1), subsequently affecting actin polymerisation and cell migration as well as neurite growth [117].

Through the phosphorylation of p27 at Ser^10^, CDK5 stabilises and increases the amount of p27, resulting in the upregulation of the non-phosphorylated form of cofilin via the suppression of RhoA [118]. This facilitates actin reorganisation in the migration activity [118]. This facilitates actin reorganization in the migration activity. CDK5 phosphorylates Wiskott-Aldrich syndrome protein-family verprolin homologous protein 1 (WAVE-1) to inhibit actin polymerisation [119]. In contrast, inhibition of the CDK5-mediated phosphorylation of WAVE-1 promotes actin polymerisation along with an increased number of dendritic spines [119]. Also, the activation of Eph receptor A4 (EphA4) phosphorylates and activates CDK5, which promotes the activation of ephexin-1. This contributes to enhanced actin dynamics [56].

### 3.3. CDK5 Cell Cycle and DNA Damage

DNA damage is an initiator of neuronal cell death and a stimulator of protein calpain, which is involved in the cleavage of p35 into p25 [120]. CDK5 has been shown to increase in the early stages of DNA damage [121]. In a neuron, through the CDK5-dependent phosphorylation of ataxia-telangiectasia mutated (ATM) protein, DNA damage induces cell death [122]. In contrast, disruption of the CDK5-ATM pathway protects the neuron from DNA damage [122].

CDK5 does not directly affect the cell cycle, but nuclear CDK5 plays a role as a cell cycle checkpoint [123,124,125]. The cell cycle is blocked when CDK5 is upregulated in the nucleus but not the cytoplasm [124]. Moreover, cells that re-enter the cell cycle have a lower abundance of nuclear CDK5 [124]. More critically, cell cycle disruption requires the binding of CDK5 to p35, but not p25 or p39 [36].

CDK5/p35 plays a role in transforming growth factor-β1 (TGF-β1)-induced cell cycle arrest at the G1 phase [126]. The inhibition or knockdown of CDK5 dramatically reduces the frequency of TGF-β1-mediated cell cycle arrest [126]. Interestingly, TGF-β1 enhances the p35-dependent activity of CDK5 [126].

However, neurons are at risk of death when they re-enter the cell cycle [36]. CDK5 can prevent cell cycle re-entry as a neuroprotective mechanism when CDK5 is activated by p27 or p35 [36,123,127]. CDK5/p27 or CDK5/p35 complexes in turn binds to E2F transcription factor 1 (E2F1) [36,127]. This disrupts the interaction between E2F1 and transcription factor DP-1, resulting in cell cycle inhibition [36,127].

In contrast, under hyperactive conditions through p25 or p35 overexpression, CDK5 phosphorylates retinoblastoma (Rb), which can re-enter the cell cycle (leading to cell death) through the CDK5-Rb-E2F pathway [128,129]. This could not occur under normal condition [129].

Moreover, the CDK5-mediated phosphorylation of casein kinase 1 (CK1) results in diminished CK1 activity [130]. CK1 plays a vital role in an array of signalling pathways, such as DNA repair, apoptosis, proliferation, and cell differentiation [130,131]. Thus, CDK5 may have an indirect effect on the cell cycle as well as cell proliferation.

## 4. The Role of CDK5 in Cancer Cells

**Figure 4:**
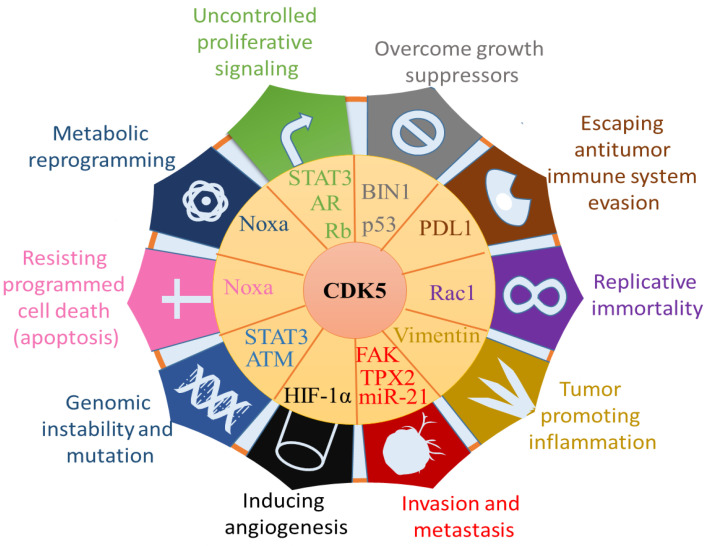
Impacts of CDK5 on the hallmarks of cancer: Uncontrolled proliferative signalling, Overcome growth suppressors, escaping antitumor immune system evasion, replicative immortality, tumour promoting inflammation, invasion and metastasis, inducing angiogenesis, genomic instability and mutation, resisting programmed cell death, metabolic reprogramming. Through the effect on the target protein, CDK5 is conducive to tumour development and metastasis. Abbreviations: phorbol-12-myristate-13-acetate-induced protein 1 (Noxa), Signal transducer and activator of transcription 3 (STAT3), androgen receptor (AR), retinoblastoma (Rb), bridging integrator 1 (BIN1), programmed cell death ligand 1 (PD-L1), focal adhesion kinase (FAK), targeting protein for Xklp2 (TPX2), hypoxia-inducible factor-1α (HIF-1α), ataxia-telangiectasia mutated (ATM). (Line 516–525). This figure is adapted and modified from Lenjisa et al., and Stecca et al. [134,135].

To date, characteristics for tumour development have been outlined. Ten features are considered to be the hallmarks of cancer [132,133]. CDK5 can be regarded as a potential target for anticancer treatment through its effects on these hallmarks (Figure 4). We present the influence of CDK5 on cancer to shed light on the potential role of CDK5 in the tumorigenesis pathway.

### 4.1. Effects of CDK5 on Cancer Hallmarks from Tumour Itself

CDK5 upregulation has been associated with a variety of cancers such as colorectal cancer (CRC), lung cancer, and nasopharyngeal cancer (NPC) [10,136,137]. Besides, high expression of CDK5 suggests a poor prognosis for CRC, lung cancer, and liver cancer along with short overall survival in lung cancer and ovarian cancer [136,137,138,139,140]. In particular, the high enzymatic activity of CDK5 was also confirmed in liver and prostate cancer [75,141]. However, some authors argued that CDK5 acts as a tumour suppressor in gastric cancer [142,143,144]. Low expression of CDK5 displays poor patient survival, while nuclear CDK5 accumulation prevents the proliferation and metastasis of gastric cancer cells [142,143]. This is associated with serine/threonine-protein phosphatase 2A (PP2A) and p27 expression [142,144].

#### 4.1.1. Effect of CDK5 on the Proliferation and Growth of Cancer

CDK5 may phosphorylate tumour suppressors and transcriptional factors. In lung cancer, CDK5 promotes the tumour’s progression by inhibiting the tumour-suppressive function of bridging integrator 1 (BIN1) [7]. When binding to c-Myc, BIN1 can suppress cell proliferation, inducing apoptosis [145,146]. However, the phosphorylation of c-Myc at Ser^62^ acts as a barrier preventing the interaction between c-Myc and BIN1 [147]. Zhang et al. showed that CDK5 could phosphorylate Ser^62^ of c-Myc, inhibiting the BIN1-c-Myc interaction and indirectly facilitating cancer proliferation [7]. It has been demonstrated that epidermal growth factor receptor (EGFR)-activated CDK5 phosphorylates and binds to tripartite motif-containing 59 (TRIM59), translocating TRIM59 into the nucleus [148]. This makes histone variant, macroH2A1, a tumour suppressor, more prone to the ubiquitination and degradation [148]. This promotes STAT3 signalling activation and tumorigenesis [148]. Moreover, CDK5 is also a positive regulator to cell proliferation through STAT3 activation and STAT3-mediated androgen receptor (AR) activation via the phosphorylation of STAT3 at Ser^727^ in prostate cancer [75]. Correspondingly, in medullary thyroid cancer (MTC), through the Ser^727^ phosphorylation of STAT3, CDK5 modulates cell proliferation [76]. According to these findings, CDK5 is a crucial regulator of cancer cell proliferation and survival.

#### 4.1.2. Effect of CDK5 on the Migration and Invasion

The migration of malignant tumours to adjacent sites and distant sites is the first step of cancer metastasis [149]. There is much evidence showing the role of CDK5 in the migration of numerous cancers, including pancreatic cancer [150], breast cancer [9], lung cancer [151], liver cancer [139], glioblastoma [152], prostate cancer [102], and pituitary cancer [153]. CDK5 downregulation marginally attenuated the migration of these cancers [102,139,150,151,152,153].

In response to epidermal growth factor (EGF), Gα-interacting vesicle-associated protein (GIV) is phosphorylated by CDK5, subsequently activating Gαi and promoting promigratory Akt signals [154]. Consequently, these events enhance cancer migration [154]. Liu et al. demonstrated that, in glioblastoma, CDK5 could activate nuclear Akt to favour the cancer migration and invasion via the phosphorylation of isoform A of phosphatidylinositol 3-kinase enhancer (PIKE-A) [152].

Tumours can migrate to second sites via epithelial-mesenchymal transition (EMT), in which carcinoma cells undergo the loss of cell polarity and cell-cell adhesion and act as mesenchymal stem cells. CDK5 is introduced to stimulate the activity of targeting protein for Xklp2 (TPX2) [139], leading to the migration of hepatocellular carcinoma cells [139,155]. TPX2 can enhance EMT via EMT-related proteins, such as E-cadherin, N-cadherin, β-catenin, Slug, MMP-2, and MMP-9 [156]. Liang et al. reported that, in breast cancer, CDK5 overexpression facilitates transforming growth factor-β (TGF-β)-induced EMT via Ser^732^ phosphorylation of FAK [9]. In contrast, the deletion of CDK5 attenuates TGF-β-induced EMT and suppresses cell motility [9]. In head and neck squamous cell carcinoma, CDK5 also plays an important role when cooperating with the miR-21 gene to facilitate EMT [157].

Blocking CDK5 activity results in failed cytoskeletal remodelling in lung cancer cells, causing them to lose the cell polarity and decline in cellular mobility [103]. Strock et al. pointed out that CDK5 inhibition leads to changes in cytoskeletal properties, cellular polarity, and invasion potential [102]. In breast cancer, while CDK5 inhibition causes the depolymerisation and the rearrangement of F-actin, CDK5 upregulation potentiates F-actin bundles [9]. Evidence also indicates that CDK5-mediated phosphorylation of actin-binding protein adducin-1 (ADD1) at Thr^724^ decreases the affinity of ADD1 with F-actin, which might, in turn, reorganise actin during cell migration [104]. Hence, the inhibition of migration factors by suppressing the CDK5 signalling pathway is an attractive strategy for preventing cancer invasion.

#### 4.1.3. Effects of CDK5 on the Genome Instability, Mutation, and Replicative Immortality

Genome instability is among the characteristics that cause mutation in DNA repair genes to lead to cancer [158]. CDK5 may be essential for the activation of intra-S and G2/M checkpoints, which are prerequisite to DNA repair [159,160]. CDK5 phosphorylates replicating protein A subunit (RPA32), which is subsequently conducive for the intra-S phase checkpoint induction and DNA repair [159]. Additionally, CDK5 can phosphorylate STAT3 at Ser^727^ so that p-STAT3 could interact with endonuclease essential meiotic structure-specific endonuclease 1 (Eme1) [161]. This facilitates the rescue of damaged replication forks [161].

In a neuron, through CDK5-mediated phosphorylation Ser^794^ of ATM, CDK5 activates tumour suppressor, p53, and modulates cell death [122]. Interestingly, in HCC, CDK5 inhibition increases DNA double-strand breaks via the presence of DNA damaging agents [141]. Relatedly, with the inhibition of CDK5, the response to DNA damaging agents of HCC cells significant increases [141].

Moreover, signs of DNA replication stress include oncogene-mediated senescence [162]. Although at an early stage, senescence can prevent malignant transformation, prolonged senescence can facilitate cancer development [163]. Mao et al. reported that Rb-dependent senescence requires the CDK5 activation by p35 [164]. Subsequently, CDK5 inhibits GTPase Rac1, which is associated with senescent cytoskeletal changes [165]. This suggests the indispensable role of CDK5 in senescence and cellular immortalisation.

#### 4.1.4. Effects of CDK5 on Cancer Cell Metabolism

There is not much evidence regarding the impact of CDK5 on cancer metabolism. However, evidence has indicated that insulin-activated CDK5 leads to the phosphorylation of extended synaptotagmin-1 (E-syt1) [166]. This facilitates the association between E-syt1 and glucose transporter type 4 (GLUT-4) so that 3T3-L1 adipocytes take up glucose [166]. Moreover, it is also demonstrated that CDK5 inhibition or knockdown inhibits the consumption of glucose [166]. Also, CDK5 regulates the secretion of insulin in response to high levels of glucose [167].

Another study revealed that, in podocytes, the expression of p35 and the kinase activity of CDK5 are driven by TGF-β1/extracellular signal-regulated protein kinases 1 and 2 (ERK1/2)/early growth response-1 (Egr-1) pathway in hyperglycaemic conditions [168]. With its function being a critical player in the proliferation, differentiation, and morphology of podocyte [169], CDK5 contributes to the development and progression of diabetic nephropathy [168].

Interestingly, with the presence of glucose, phorbol-12-myristate-13-acetate-induced protein 1 (Noxa) is phosphorylated at Ser^13^ by CDK5, subsequently inhibiting the pro-apoptotic function of Noxa and modulating glucose metabolism in the haematopoietic lineage [170]. CDK5 knockdown or the hypoglycemia lead to Noxa dephosphorylation, initiating Noxa-mediated apoptosis [170]. More importantly, Noxa stimulates glucose uptake and turns over glucose through the pentose phosphate pathway (PPP) to promote cell growth [170].

### 4.2. Effects of CDK5 on Tumour Microenvironments

Tumour microenvironments (TME) support the growth of tumour cells or hinder anti-cancer agents from accessing to tumour cells [171,172]. Thus, research on TME and factors influencing TME is essential. Also, various cells, including macrophages and fibroblasts, constitute the TME. Cancer-associated fibroblasts (CAFs), a constituent of TME, is a vital contributor to proliferation, migration, invasion, and angiogenesis [173,174,175]. However, little research has been done on the role of CDK5 in CAF. In CAFs, it has been reported that CDK5 is activated in the HOTARI-induced EMT via accumulation of trimethylation of H3K27 in the promoter region of CDK5RAP1 (CDK5 Regulatory Subunit Associated Protein 1), the suppressor of CDK5 [176,177].

Among the hallmarks of cancer, inducing angiogenesis, tumour-promoting inflammation, and the blockade of immune destruction are associated with the TME, and we describe the effects of CDK5 on these hallmarks. Also, we add the effects of CDK5 on cancer-nerve connection since nerve contribution can be one of the emerging TME.

#### 4.2.1. Effects of CDK5 on Angiogenesis

Angiogenesis is a multi-step process in which new blood vessels involving endothelial cells grow from the pre-existing blood vessels [92]. Tumour angiogenesis is the proliferative penetration of blood vessels into cancerous tissues to supply nutrients and oxygen [178]. CDK5 is involved in the proliferation and migration of endothelial cells [179].

In comparison to quiescent cells, CDK5 significantly expresses higher in proliferating cells. CDK5 induces the formation of lamellipodia to promote cell migration [179]. Accordingly, pleiotrophin (PTN) is a secreted growth factor that stimulates human endothelial cell migration by binding to receptor proteins tyrosine phosphatase beta/zeta (RPTP β/ζ) and ανβ3 integrin. CDK5 mediates the PTN-induced movement of endothelial cells [180,181]. CDK5 also promotes the stabilisation and transcriptional activity of hypoxia-inducible factor-1α (HIF-1α) through the phosphorylation of HIF-1α at Ser687, thus accelerating the formation of blood vessel [182]. CDK5 is also involved in controlling lymphatic development and function by phosphorylation of Foxc2 [183]. These facts suggest that CDK5 is an important target in regulating angiogenesis.

Treatment with CDK5 inhibitor suppresses the growth and induces the apoptosis of bovine aortic endothelial cells and human aortic endothelial cells [184]. CDK5 inhibitor also reduces migration of the endothelial cells [179]. Moreover, CDK5 knockdown diminishes DLL4-induced Notch downstream target expression and the active Notch intracellular domain generation [185]. The suppression of DLL4-Notch pathway prevents the development of tumour through the facilitating non-productive angiogenesis and preventing tumour vasculature. The ablation of CDK5 suppresses angiogenic processes in hepatocellular carcinoma (HCC) [182]. In line with this, CDK5 inhibition resulted in the reduced expression of vascular endothelial growth factor (VEGF), a vital protein for the initiation of signalling cascades responsible for angiogenesis [186].

#### 4.2.2. Effects of CDK5 on Inflammation and Immune Evasion

Inflammation contributes to tumour development [187,188,189]. Firstly, chronic inflammation can produce reactive oxygen and nitrogen species, which cause genetic damage [190]. Inflammation response processes recruit inflammatory cells, including cytokines, chemokines, and enzymes [191]. These cells establish inflammatory microenvironment to cancer development, promotion, progression, and invasion [190]. There are limited evidence regarding the impact of CDK5 on inflammation related to cancer development. However, it has been shown that CDK5-mediated phosphorylation of Ser^56^ vimentin promotes the GTP-dependent secretion of pro-inflammatory molecules by neutrophils [192]. This secretion can promote inflammation, leading to tumour development and metastasis [193]. In line with this, in melanoma, CDK5 could directly phosphorylate vimentin at Ser^56^ [194]. The decreased kinase activity of CDK5 leads to blocking the metastasis by losing both phosphorylation form of vimentin and soluble vimentin [194]. This suggests that CDK5 can regulate inflammation factors conducive to the development of cancer.

Immune evasion is among the significant mechanisms inhibiting the effectiveness of anticancer drugs [195]. For overcoming immune surveillance, tumours induce diverse mechanisms such as down-regulating antigen factors, tolerant strategies, and generating several immunosuppressive cytokines [195]. CDK5 can regulate the expression of programmed cell death ligand 1 (PD-L1) to prevent antitumour immunity [196]. CDK5 silence results in the phosphorylation of interferon regulatory factor 2 binding protein 2 (IRF2BP2), which in turn increases the expression of interferon regulatory factor (IRF2) and declines the expression of PD-L1 [196]. Through the depletion of PD-L1, CDK5 deficiency can promote CD4^+^ T-cell-mediated cancer cell death [196]. Besides, evidence also showed the role of CDK5 in T cells through its effect on histone deacetylases (HDACs) and Foxp3 gene expression [197,198]. The disruption of CDK5 activity reduces the IL-2 expression through increased activity of HDACs and suppresses the binding of STAT3 to Foxp3 gene promoter through decreased phosphorylation STAT3 at Ser^727^ [197,198]. This, in turn, regulates the differentiation and survival of T-cells [197,198]. Hence, further studies are needed to confirm the effect of CDK5 on immune, especially in cancer.

#### 4.2.3. CDK5-Nerve and Cancer Connection

Nerves are an essential part of the tumour microenvironment and contribute to tumour progression [199,200]. For example, almost peripheral cancer types have been observed to interact with nerve structures at least at an advanced stage, especially in bladder cancer, prostate cancer, pancreatic cancer, colon cancer, lung cancer, head and neck cancer, and bile duct cancer [201]. Stress-induced sympathetic activation promotes cancer, and a decrease in tumour innervation indicates a higher survival rate without recurrence [202,203]. The increase in chemotherapy response by β-blockers is due to anticancer and antiangiogenic activity [204]. As you can see from these examples, the hypothalamic-pituitary-adrenal axis (HPA) and autonomic nervous system (ANS) release neurotransmitters that bind to their receptors and contribute their functions in diverse tissues, including tumour tissue [205]. This, in turn, exerts effects on tumour growth and metastasis [205]. Indeed, matrix metalloproteinases (MMPs) are involved in the degradation of ECM proteins, the activity of cytokines, and the production of growth factors. Thus, the HPA axis and ANS serve as regulators of metastasis [206,207].

Shapiro and Warren have indicated that nerve fibres are found in several cancer tissues [205,208]. Moreover, CDK5 and p35 are correlated to nerve fibres outgrowth [209]. This suggests that CDK5 can be involved in nerve fibres-mediated cancers. Glial cell line-derived neurotrophic factor (GDNF) is an essential factor for neuronal proliferation [210]. In pancreatic cancer, GDNF can positively regulate the expression and activity of MMP-9 to facilitate cancer invasion [211]. Another neurotrophic factor, NGF, released from neuronal tissue, increases the amount of MMP-2 and the activity of MMP-2, subsequently promoting invasiveness [212]. Interestingly, in the neuron, GDNF serves as a chemoattractant factor, which increases the activity of CDK5 to facilitate the migration of rostral migratory stream cells [213].

Catecholamine hormones such as norepinephrine and epinephrine, modulate tumour development through MMPs and VEGF, a pro-angiogenetic factor, in nasopharyngeal cancer [214]. Additionally, norepinephrine and epinephrine are stress-related mediators, and they promote the release of VEGF through β-adrenoreceptor to control the process of angiogenesis in ovarian cancer [215]. More importantly, CDK5 is also a crucial protein for neuronal function via the control of catecholamine neurotransmitter release [216], neurotransmitter synthesis [216], and Munc18-mediated exocytosis [32]. CDK5 may modulate tumour development and metastasis through neurotransmitters. Although there is not much evidence showing the association between CDK5 and cancer via neurotransmitters, this can be a potential target for cancer treatment.

Therefore, CDK5 seems to be able to promote cancer progression through its role in the nerves surrounding the tumour in addition to its role in cancer cells, and further research is needed.

## 5. Potential Therapeutic Options

**Figure 5:**
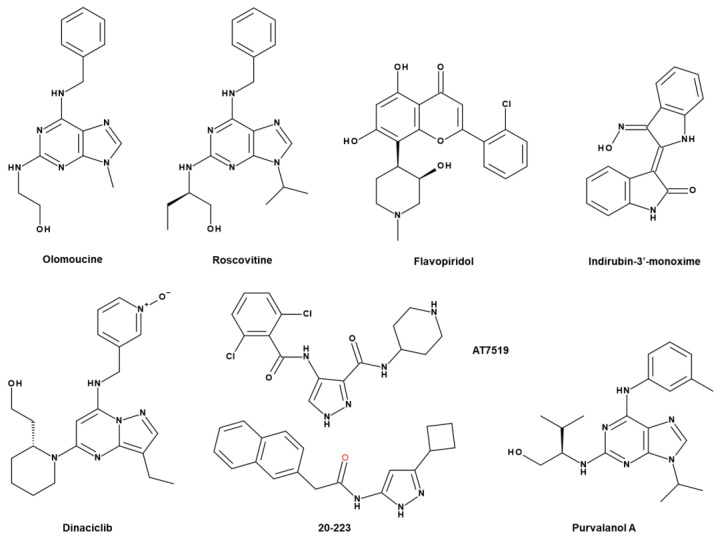
Structures of CDK5 inhibitors: roscovitine, flavopiridol, dinaciclib, olomoucine, purvalanol A, indurubin-3′, 20-223, AT7519.

To date, there are many reports regarding the CDK inhibitors, but highly selective inhibitors of CDK5 are not available. We classify the CDK5 inhibitors as early pan CDK inhibitors, multitarget CDK5 inhibitors, and selective CDK5 inhibitors according to report of Whittaker et al.’s (Figure 5) [217].

### 5.1. Early Pan CDK Inhibitors

Olomoucine, roscovitine, and flavipiridol belong to broad pan CDK inhibitors (Figure 5) [218]. Olomoucine is a selective CDK5 inhibitor [218,219]. It inhibits CDK1 (IC_50_ = 7 μM), CDK2 (IC_50_ = 7 μM) and CDK5 (IC_50_ = 3 μM) (Table 4) [218]. Olomoucine induces apoptosis in human cancer [218,220]. The combination of olomoucine with androgen-antagonist bicalutamide exerts the synergic effect on prostate cancer cell lines [221].

Among the first CDK inhibitors, roscovitine (seliciclib) and flavopiridol (alvocidib) have entered the clinical trial phase [217]. Roscovitine [CY-202, (*R*)-roscovitine, seliciclib] is a small molecule that inhibits cyclin-dependent kinase (CDK) through direct competition with ATP at the ATP binding site [227,228,229]. It is a broad-spectrum purine inhibitor that inhibits CDK1, CDK2, CDK5, and CDK7 (IC_50_ = 0.2–0.5 μM), but a weak inhibitor for CDK4 and CDK6 (IC_50_ > 100 μM) (Table 4). Also, only a few kinases such as CaMK2, CK1α, CK1δ, DYRK1A, EPHB2, ERK1, ERK2, FAK, and IRAK4 in the 1–40 µM range are sensitively inhibited by roscovitine [227]. In certain cancers such as breast cancers, roscovitine (100 mg/kg) can decrease tumour development and drug resistance in a xenograft model [230].

Flavopiridol (alvocidib), which has orphan drug designation in chronic lymphocytic leukaemia (CLL) from the FDA and the EMA [231], can inhibit the activity of CDKs, which in turn prevents tumour proliferation and facilitates apoptotic process [232,233]. Flavopiridol inhibits several CDKs such as CDK1, CDK2, and CDK4 and inhibits CDK5/p25 formation [234]. Flavopiridol induces cell cycle arrest in NSCLC cells and apoptosis in oral cancer cells [235,236].

Indirubin-3′-oxime is also a potent inhibitor of CDK5/p25 (IC_50_ = 0.10 μM) and GSK3β (IC50 = 0.022 μM) (Figure 5) [237]. Indirubin-3′-monoxime represses tumour proliferation, along with a low concentration of phosphorylated-CDKs in the nucleus [238]. Moreover, indirubin-3′-monoxime inhibits the migration and invasion of pancreatic cancer cells through the downregulation of MMP-9 [238]. Indirubin-3′-monoxime inhibits tumour formation of oral cancer through downregulation of survivin at 10 μM [239].

### 5.2. Multitarget CDK5 Inhibitors

Dinaciclib (SCH 727965) is a small molecule multi-CDK inhibitor targeting CDK 2/5/9 with an improved therapeutic index in comparison with flavopiridol (Figure 5) [223]. Through restraining Rb phosphorylation, dinaciclib leads to apoptosis in tumour cell lines and reduces tumour volume in xenograft mode at 48 mg/kg [223]. In the phase I study, dinaciclib, combined with rituximab, was well tolerated and revealed encouraging clinical activity in relapsed/refractory chronic lymphocytic leukaemia patients [240]. As a single agent, it showed a positive treatment effect in patients with relapsed multiple myeloma [241].

AT7519 was discovered through a structure-guided, fragment-based, screen and AT7519 inhibits CDK1 (IC_50_ = 0.21 μM), CDK2 (IC_50_ = 0.047 μM), CDK4 (IC_50_ = 0.1 μM), CDK5 (IC_50_ = 0.13 μM), CDK6 (IC_50_ = 0.17 μM), and CDK9 (IC_50_ = 0.13 μM) (Figure 5; Table 4) [242]. AT7519 inhibits the growth and promotes the death of paclitaxel-resistant cervical cancer cells and 5-fluorouracil-resistant colon cancer cells [243]. This suggests the potential role of AT7519 in drug resistance.

Like AT7519, 20-223 (CP668863) is a CDK inhibitor derived based on the 4-aminopyrazole core (Figure 5) [225,242]. 20-223 was more potent than AT7519, and 20-223 was equipotent against CDK2 and CDK5 compared to other CDK family members [225]. Treatment with 20-223 (CP668863) decreases tumour growth as well as the weight and volume of tumours in xenograft model of colorectal cancer [225]. Moreover, compared to control, HCT116 cells decrease migration upon treatment with 20-223 [225].

### 5.3. Selective CDK5 Inhibitors

There seems to be no specific inhibitor for CDK5 yet. Purvalanol A was derived by a combinatorial chemistry approach as a selective inhibitor for CDK2 (IC_50_ = 4–70 nM) and CDK5 (IC_50_ = 75 nM) (Figure 5; Table 4) [218]. Through cell cycle arrest, purvalanol A is mentioned as an apoptotic inducer in various cancers, such as breast and prostate cancer [244,245,246]. Some purvalanol A-related pathways have been reported, such as the activation of polyamine catabolic pathway and natural polyamines catabolic pathway [245,246].

Recently, TFP5/TP5, a peptide derived from p35, was found to inhibit the hyperactivity of CDK5/p25 complex without influencing endogenous CDK5/p35 complex [247,248,249]. In glioblastoma, TP5 reduces cell viability and growth through the prevention of ATM phosphorylation [247]. TP5 is synergistic with the current standard of cancer care in the treatment of glioblastoma [247,250,251].

Although there are various types of CDK5 inhibitors and researches are ongoing to develop selective CDK5 inhibitors [252], specific CDK5 inhibitors are not yet available for chemotherapy in the clinical area. Thus, further studies and efforts for specific CDK5 inhibitors are needed.

## 6. Perspectives

We have presented a summary of CDK5 as a biomarker and a new target in cancer treatment. CDK5 is overexpressed in various cancer, and this upregulation has been proposed to facilitate tumour proliferation and metastasis. There is much research regarding the effect of CDK5 on cancer hallmarks. However, the effects of CDK5 on some hallmarks have not yet been studied.

Notably, CDK5 is involved in the metabolic processes associated with glucose consumption and facilitating cell proliferation. Glucose is known as an essential nutrient for cancer development. This raises the question of whether CDK5 manipulates tumour development through glucose uptake. This novel role of CDK5 needs to be studied further.

CDK5 was first known as a kinase regulating neuronal function. Recently, evidence has shown the involvement of CDK5 in cancer. Therefore, among the several findings that have been studied in neurons, for example, the roles of CDK5 related to the reorganisation of cytoskeletons, including microtubules, are likely to apply to cancer research. CDK/p35 is activated in several cancers and promotes proliferation and migration of cancer cells. However, other groups reported that CDK5 inhibits proliferation and migration of cancer cells. Recently, Sharma et al. have utilised the melanoma cells expressing analogue-sensitive CDK5, which made it possible to specifically inhibit CDK5 in cancer cells, clearly demonstrating that inhibition of CDK5 in melanoma cells inhibits the metastatic spread of melanoma [194].

If the researchers’ approach can be applied to various carcinomas involving CDK5, clear conclusions can likely be drawn from the conflicting results associated with CDK5 in different cancers. If it becomes clear that CDK5 promotes cancer development and progression, the development of CDK5 specific inhibitor is expected to accelerate further. Since the research results on inhibitors of CDK5 have been conducted to develop therapeutics for neurological diseases, if these results can be used to develop therapeutics for various cancers, it is expected to create a specific inhibitor for CDK5 at a relatively rapid pace. Besides, since CDK5 is a target present in nerves, the neurological side effects of concern can be minimised through a strategy that prevents the passage of the blood-brain barrier, and a study of CDK5 therapy in carcinomas where nerves are involved in the growth and progression of cancer. In conclusion, CDK5 is a fascinating target in cancer. We look forward to the day that the development of CDK5 specific inhibitors through many studies will be successful, and it will become a therapeutic agent for various refractory cancers.

## Figures and Tables

**Table 1:** The regulatory subunits of CDK5.

Protein	Position	Interaction with CDK5
p35/p25	p35 mainly localises in the plasma membrane, perinuclear region, and less in the nucleus [23], whereas p25 mostly exists in the cytosolic region and nucleus [24].	p35 can activate CDK5 through binding to CDK5 [19]. However, the binding with p25 leads to the hyperactivation of CDK5 [24].
p39/p29	p39 localises in the plasma membrane and nucleus [25].	p39/p29 can bind to CDK5 and then activate CDK5 [25].
Cyclin I		Cyclin I could activate CDK5 by forming a complex with CDK5, and this complex acts as an anti-apoptotic factor [26].
Cyclin D1	During G1 phase, cyclin D1 is synthesised and localises in the nucleus before entering S phase [27].	Cyclin D1 competes with p35 to inhibit CDK5, contributing to neuronal apoptosis through the MEK-ERK pathway [28].
Cyclin E	All cell cycle phase, cyclin E is synthesised and accumulated in the nucleus [29].	Cyclin E binds to CDK5 to prevent the interaction between CDK5 and its activators, leading to effects on synapse function and memory [30].
GSTP1		GSTP1 inhibits the activity of CDK5 through two mechanisms: competing with p35 or p39 to bind to CDK5; depleting oxidative stress [22].
Munc18		Munc18 binds to and protects the CDK5/p35 complex from the inhibitory effect of TFP5 [31,32].

**Table 2:** The regulation of CDK5 by posttranslational modification.

Site	Effect	Ref.
Phosphorylation		
Tyr15	Facilitates the activity of CDK5.	[54,55,56,57]
Ser159	Is required for maximal activation of CDK5/p35 complex. Facilitates the activity of CDK5/p25 complex.	[58,59]
Ser47	Suppresses the interaction between CDK5 and p35, leading to decreasing kinase activity of CDK5 and interfering with cell migration.	[60]
Thr14	Inhibits the activity of CDK5.	[61]
Thr77	Disrupts the interaction between CDK5 and p35, resulting in the inactivation of CDK5.	[62]
	S-nitrosylation	
Cys83	Inhibits the activity of CDK5.	[63,64]
Cys157	Inhibits the activity of CDK5.	[63,64]
	Acetylation	
Lys33	Inhibits the activity of CDK5.	[65]

**Table 3:** The modulation of transcription factors by CDK5.

Transcription Factor	CDK5 Complex	Phosphorylation Site	Physiological Significance	Ref.
MEF2	CDK5/p25	Ser^444^	Neuronal cell death	[70,71,72,73]
STAT3		Ser^727^	Cancer	[74,75,76,77]
MR	CDK5/p35 CDK5/p25	Ser^128^ Ser^250^ Thr^159^	Neuron function	[78]
GR	CDK5/p35	Ser^203^ Ser^211^	Neuron function	[79]
	CDK5/p25			
p53	CDK5/p35	Ser^15^ Ser^33^ Ser^46^	Neuronal cell death	[80,81]
TonEBP/OREBP		Thr^135^	Osmotic stress.	[82]
MEK1	CDK5/p35	Thr^286^	Cell death	[83]
mSds3	CDK5/p35	Ser^228^	Neuron and muscle development.	[84]

**Table 4:** IC_50_ values of CDK5 inhibitors.

Inhibitor	IC_50_ (µM)	Ref.
Roscovitine	0.16	[222]
Flavopiridol	0.014	[223]
Dinaciclib	0.001	[223]
Olomoucine	3	[222]
Purvalanol A	0.075	[218]
Indirubin-3′-monoxime	0.1	[224]
20-223	0.0088	[225]
AT7519	0.011–0.013	[226]

## Data Availability

Data sharing not applicable.
